# Using Canonical Correlation Analysis to Discover Genetic Regulatory Variants

**DOI:** 10.1371/journal.pone.0010395

**Published:** 2010-05-13

**Authors:** Melissa G. Naylor, Xihong Lin, Scott T. Weiss, Benjamin A. Raby, Christoph Lange

**Affiliations:** 1 Department of Biostatistics, Harvard School of Public Health, Boston, Massachusetts, United States of America; 2 Channing Laboratory, Brigham and Women's Hospital, Boston, Massachusetts, United States of America; Albert Einstein Institute for Research and Education, Brazil

## Abstract

**Background:**

Discovering genetic associations between genetic markers and gene expression levels can provide insight into gene regulation and, potentially, mechanisms of disease. Such analyses typically involve a linkage or association analysis in which expression data are used as phenotypes. This approach leads to a large number of multiple comparisons and may therefore lack power. We assess the potential of applying canonical correlation analysis to partitioned genomewide data as a method for discovering regulatory variants.

**Methodology/Principal Findings:**

Simulations suggest that canonical correlation analysis has higher power than standard pairwise univariate regression to detect single nucleotide polymorphisms when the expression trait has low heritability. The increase in power is even greater under the recessive model. We demonstrate this approach using the Childhood Asthma Management Program data.

**Conclusions/Significance:**

Our approach reduces multiple comparisons and may provide insight into the complex relationships between genotype and gene expression.

## Introduction

The usefulness of examining associations between genetic markers and gene expression is due to the immediate and direct relationship between the gene expression phenotype and DNA sequence variation. As Rockman and Kruglyak stated, “The road from genotype to phenotype runs through gene expression” [1].

Most studies of transcriptional regulation have relied on univariate tests to find significant associations, each between a single genetic marker and a single expression probe. In genomewide association scans, one can easily imagine having a million single nucleotide polymorphisms (SNPs) and thousands of expression probes. The number of tests required to search for associations between individual SNPs and individual probes severely reduces power.

Canonical correlation analysis (CCA) is a statistical method that can reduce the number of tests by using multiple phenotypes and genotypes in each test. CCA compares two sets of variables (in this case, a set of SNP genotypes and a set of expression levels) to assess the correlation between them [2]. CCA finds a linear combination of the genotypes and a linear combination of the expression levels such that the correlation between the two is maximized. As it is, CCA cannot be applied to all SNPs and expression probes in a genomewide association study since the number of variables is greater than the number of subjects. Two modifications of CCA have recently been proposed for use with genetic marker and gene expression data: penalized CCA [3] and sparse CCA [4]. These methods are computationally intensive and are sometimes sensitive to starting parameters. Futhermore, they have not been simulated or applied to datasets as large as a genomewide association study. Here, we assess the power of a more straightforward approach: partitioning the data so that CCA can be applied to each subset of the data. CCA is used to construct one association test for a group of gene expression traits and a group of SNPs, thus reducing the burden of multiple comparisons. Using CCA in this way, we can not only pick out regions of the genome in which genotype is highly associated with gene expression, but can also potentially discover more complex relationships among the variables by examining the coefficients of the linear combinations with maximum correlation. We examine the potential of CCA to assess correlation between SNP data and expression data using simulations and a data analysis.

## Methods

### Canonical Correlation Analysis

In the 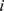
th proband, 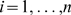, we denote the 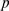 expression measurements to be used in a single test by 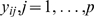, and the 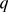 genotypes at the 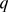 SNPs by 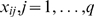. The corresponding matrices of expression profiles and genotypes are given by 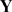 and 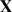. Without loss of generality, we assume the number of measured profiles, 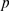, is less than or equal to the number of SNPs, 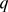. (If not, the notation for profiles 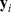 and the genotypes 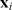 can be switched.) 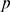 and 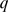 must each be less than the number of subjects, 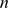.

We denote the 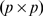 covariance matrix of gene expressions by 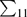, the 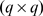 covariance matrix of genotypes by 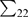, and the 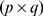 covariance of gene expressions and genotypes by 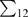. 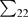 can be thought of as a measure of the linkage disequilibrium among SNPs. In the application, all three variance/covariance matrices are obtained from their empirical estimators based on 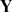 and 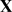. Note that 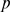 and 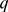 must be chosen to be small enough to ensure that the covariance matrices are not nearly singular.

The maximum correlation 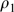 between the set of expression profiles and the set of SNPs is given by the maximum eigenvalue of the matrix 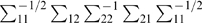 where 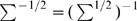 [5]. The weights for the linear combination of the expression profiles and for the combination of marker scores are given by 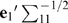 and 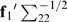, respectively, where the vector 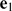 is defined to be the eigenvector associated with the maximum eigenvalue of 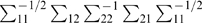 and 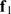, the eigenvector associated with the maximum eigenvalue of 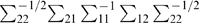.

This yields the maximally correlated linear combinations, often referred to as the first pair of canonical variates:
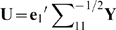
and
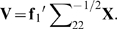
Further canonical variates that maximize correlation between two linear combinations, under the constraint that they are independent of all preceding canonical variates, can easily be calculated. The 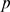 eigenvalues of 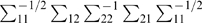, 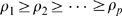, are the cannonical correlations for the 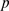 sets of canonical variates.

Several tests have been developed to assess whether the two linear combinations derived using CCA are truly correlated. Bartlett proposed the following likelihood ratio test of 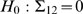 versus 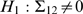, i.e., whether the covariance between the gene expression and genotypes is zero [6]:
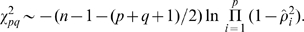
This is equivalent to testing whether all the canonical correlations equal zero.

### Simulation Methods

To apply CCA in our simulations, we chose a region of the genome containing three genes and picked out 20 tag SNPs to simulate. Using CCA, we found two linear combinations, one of the expression profiles and one of the SNP data, such that the correlation between the two linear combinations is maximized. The observed correlation between the two sets was then tested for association, using the Bartlett test statistic.

To simulate realistic genotypes, we mimicked the linkage disequilibrium structure of 20 tag SNPs in the HapMap CEU sample from a 350 kb region of chromosome 2 (positions 118250000–118600000, NCBI build 36, Figure S1). Tag SNPs were chosen such that each SNP not chosen had 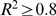 with at least one tag SNP in the HapMap CEU sample. To do this we used Haploview software v3.32 [7]. To estimate the linkage disequilibrium structure between each pair of consecutive SNPs, we estimated the haplotype frequencies for each pair of consecutive SNPs using the expectation-maximization (EM) algorithm implemented in the haplotype procedure of SAS/Genetics™ software.

We simulated the allele of the first SNP as a Bernoulli random variable with probability equal to the SNP's estimated allele frequency. Each consecutive allele in the 20 SNP haplotype was simulated conditional on the previous SNP's allele (using the conditional probabilities from the SAS haplotype procedure). 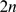 haplotypes were simulated.

Three genes lie in the chromosome 2 region: DDX18, CCDC93 and INSIG2. To determine a realistic correlation structure for the expression levels of these three genes, we examined microarray expression data from a study on aging done at Children's Hospital of Boston [8]. The covariance matrix,
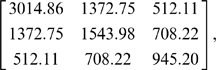
and mean expression levels,
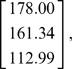
were then used to simulate expression levels for three genes assuming a multivariate normal distribution.

Simulations assuming varying heritabilty of the expression trait, sample size (200 or 400 individuals), and genetic model (additive or recessive) were run to assess type one error rate and power. For each combination of heritability, sample size, and genetic model, at least 10,000 replications were run with 20 SNPs and three gene expression levels generated for each individual each time. CCA was performed using the cancor function in R [9], which standardizes the variables in order to reduce the chance that the magnitude of the coefficients would be unduly influenced by variables with larger magnitudes. We first simulated independent SNP and expression data to determine the size of Bartlett's likelihood ratio test for no correlation between the two variables. To model an association between a single SNP and a single gene we allowed the mean expression level to vary according to the genotype of a single SNP. Gene expression levels were modeled as multivariate normal with covariance equal to the covariance matrix from the Children's Hospital Boston data. The mean was set equal to zero for genes unaffected and equal to the genetic effect size times a function of the genotype for affected genes. For the additive genetic model, the effect size was multiplied by the number of copies of the risk allele. For the recessive model, the effect size is multiplied by two if and only if there are two copies of the risk allele. Simulating a recessive model is equivalent to simulating a dominant model using the opposite allele. Since we were not interested in simulating a signal between any particular SNP and gene, we randomly chose which SNP and gene would be correlated in each replication, giving all possible pairs equal probability. All simulations were performed in R v2.2.1 [9].

## Results

### Simulation Results

Under the null hypothesis, the estimated probability of a type one error using Bartlett's likelihood ratio test decreases with increasing sample size (Table 1). For sample sizes greater than 60, the type one error is near 5%. Under the alternative hypothesis of association, the power to detect a significant correlation with Bartlett's test is compared with the power to detect the simulated association by regressing the expression quantitative trait locus (eQTL) of interest on the number of copies of the risk allele using Bonferroni correction to adjust for 60 pairwise tests (the number of pairwise tests needed to test each of the three eQTLs with each of the 20 SNPs) (Table 2). The type one error of the univariate test, 0.2%, is lower than that of CCA because the Bonferroni correction is overly conservative.

**Table 1:** Type one error.

Sample Size	Type I Error
30	0.124
40	0.068
50	0.057
60	0.054
100	0.047
200	0.047
400	0.049
500	0.049
1000	0.048

Type one error of Bartlett's test for correlation between 20 SNPs and three gene expression traits simulated under the null hypothesis of no correlation.

**Table 2:** Power.

Sample Size	Genetic Model	Analysis	Heritability								
			0	0.03	0.06	0.09	0.12	0.15	0.18	0.21	0.24
400	additive	CCA	4.9	54	91	99	100	100	100	100	100
		regression	0.2	56	95	100	100	100	100	100	100
	recessive	CCA	5.0	18	35	49	58	65	68	72	74
		regression	0.2	12	34	50	60	66	71	75	77
200	additive	CCA	4.5	25	53	77	92	97	99	100	100
		regression	0.2	19	57	84	96	99	100	100	100
	recessive	CCA	4.9	11	18	25	34	40	47	52	56
		regression	0.2	4	14	24	35	44	52	57	60

Estimated percent power of Bartlett's test and univariate regression after Bonferroni correction. (Genetic model is not applicable when heritability is zero because no genetic effect is simulated.)

**Figure 1:**
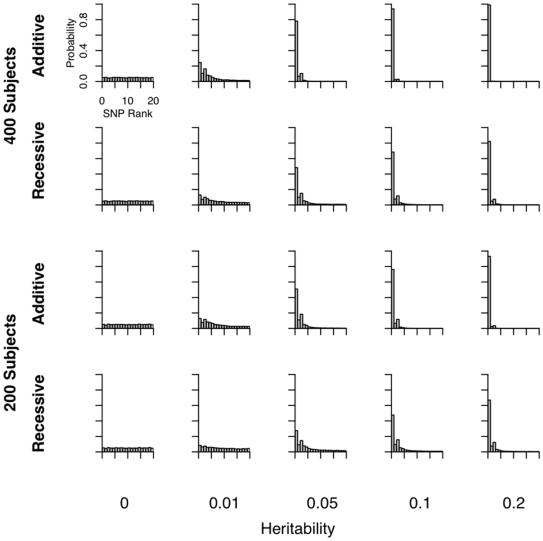
Histograms of the rank of the SNP of interest in simulations. Each panel represents a distinct set of simulations conditions: number of subjects, genetic model, and heritability. For each simulated dataset in which there was a significant correlation between SNPs and gene expression traits (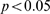 for Bartlett's test), SNPs were ranked according to the magnitude of their coefficients in the top canonical variate. Each panel is a histogram of ranks of the SNP of interest. The first column shows that the rank is uniformly distributed under the null hypothesis. The axis labels on the upper left plot apply to all plots.

**Figure 2:**
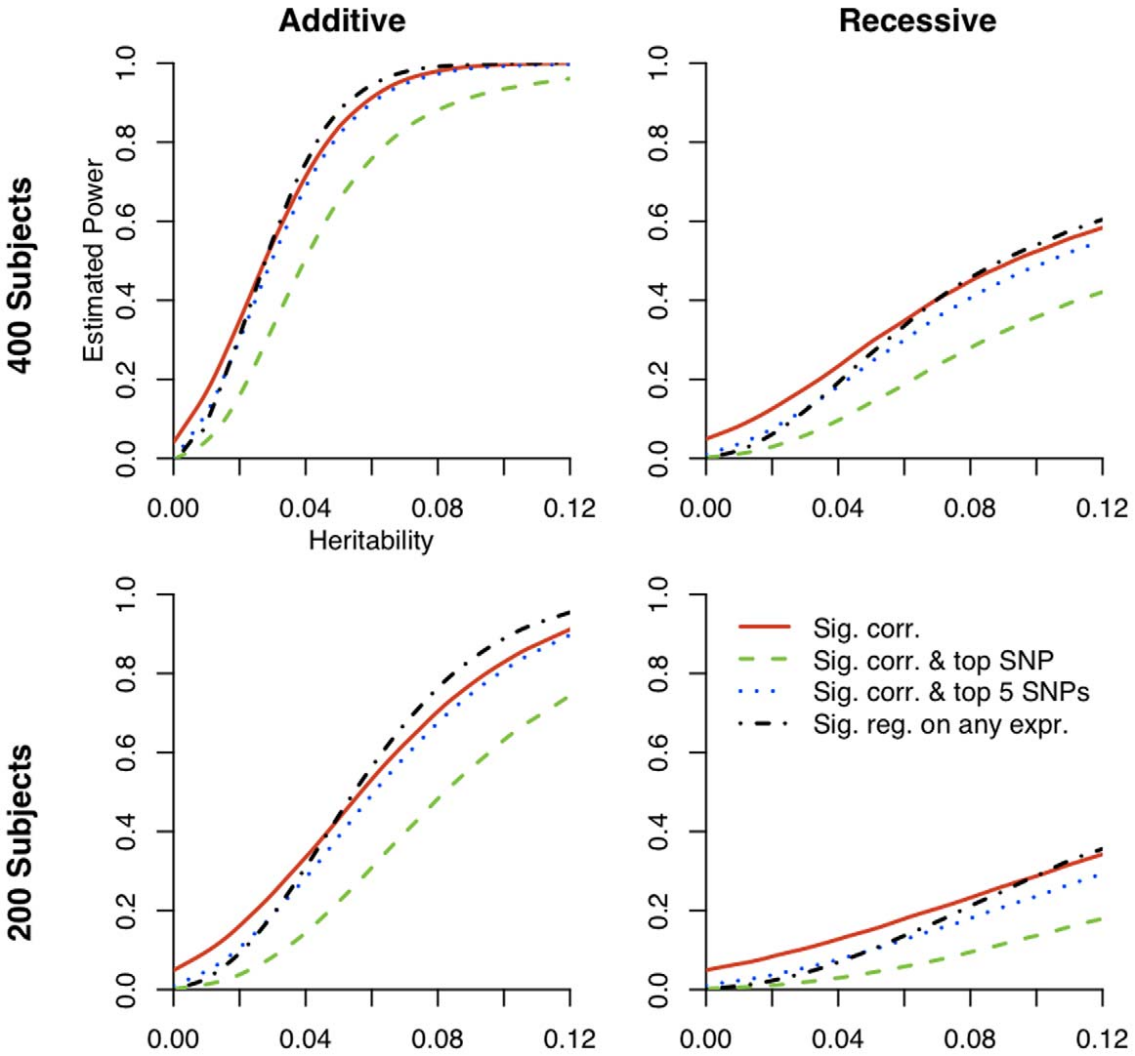
Power for three methods of determining a significant finding. The red line shows power to detect a correlation in the simulated region via Bartlett's test with 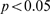. The blue line shows power to detect a correlation and have the SNP of interest be in the top five ranking SNPs (based on magnitude of SNP coefficients). The green shows power to detect a correlation and have the SNP of interest be the top ranking SNP. The black line shows power to detect an association between the SNP of interest and at least one of the three genes using univariate regression.

We assessed the effectiveness of CCA by considering both the power to detect an association between the SNPs and eQTLs and the ranking of the magnitude of the coefficient of the SNP of interest relative to the coefficients of all non-associated SNPs (Figure 1). More specifically, we considered the power to detect a correlation (using Bartlett's test) and have the SNP of interest be the top ranking SNP or be in the top five ranking SNPs (Figure 2). When the heritability of the eQTL is moderately high (i.e., greater than 0.10) both CCA and univariate regression have high power to detect association, with univariate regression slightly out-powering CCA. For eQTLs with low heritability, CCA has greater power than univariate regression in our simulations. This effect is even more pronounced for the smaller sample size (200 individuals) and for the recessive model.

### Data Analysis - Childhood Asthma Management Program

To apply CCA to a genomewide association study, we partitioned the expression data into groups of three consecutive probes and then tested for association between the probes and SNPs that fell within the window spanned by the three probes and a 200 kb margin on each side.

We applied CCA to a subset of the data from the Childhood Asthma Management Program (CAMP), a multi-center, randomized, clinical trial involving 1,041 children with asthma [10]. The subset consisted of 156 children with CD4+ peripheral blood lymphocyte gene expression data and genomewide SNP genotype data (Infinium II 550K SNP array) available. Although complete trios were available in this dataset, we only used the offspring data to mimic a population-based design.

We searched for local regulatory variants by partitioning the probes (measuring gene expression) into sets of three and considering all SNPs located within 200kb from the ends of the two outer probes. CCA was performed with a maximum of 20 SNPs per group and also with a maximum of 50 SNPs per group. For many probe trios there were more than the maximum number (20 or 50) of SNPs in the region. In these cases, we decreased the number of probes used until the number of SNPs in the region was low enough. If there were too many SNPs for a single probe, then the SNPs were divided into groups such that each group was as close as possible to the maximum number of allowed SNPs. SNPs were always partitioned by genomic position. For each group of SNPs and nearby gene expression traits, we applied Bartlett's likelihood ratio test. For each test significant after Bonferroni correction, we examined the canonical variates (Table 3). As a comparison, each probe-SNP pair was tested for association using univariate regression (any SNP located within 200kb of the end of the probe was considered).

**Table 3:** Canonical correlation analysis of Childhood Asthma Management Study with maximum number of SNPs (ms) equal to 20 and 50.

	Univariate	All Tests	All Tests	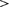 1 probe	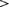 1 probe
	Regression	ms = 20	ms = 50	ms = 20	ms = 50
Total Number of tests	1759512	97773	42667	156	1488
Number of tests significant at the 0.05 level after bonferroni correction	1749	412	177	6 (10)	14 (17)
Number of SNPs in a significant CCA test with non-zero coefficient in the top canoncical variate		6434	5669	151	463
Number of SNPs significant in both a univariate regression and in a CCA test		908	575	23 (25)	53 (62)
Number of SNPs significant in a univariate regression and ranked number one in a significant CCA test		177	70	6 (9)	10 (13)
Number of SNP-probe pairs significant in a univariate regression and ranked number one probe and SNP in a significant CCA test		174	70	6 (8)	10 (13)
Number of CCA tests w/3 probes		107	1138		
Number of CCA tests w/2 probes		49	350		
Number of CCA tests w/1 probes		97617	41179		

If two numbers are listed then the second number is only adjusted for the number of tests with more than one probe.

For the analysis with a maximum of 20 SNPs per group, 908 of the SNPs found significant using univariate regression were located in a group of SNPs that was significantly correlated using CCA. Using a maximum of 50 SNPs, there were 575 such SNPs. Although the number of tests significant after Bonferroni correction was much lower for CCA than for univariate regression (412 for 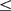
20 SNPs or 177 for 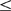
50 SNPs vs. 1749), the number of SNPs that were in a significant region was very high (6434 for 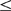
20 SNPs or 5669 for 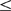
50 SNPs).

### About the Data

The Childhood Asthma Management Program (CAMP) was a 4.5 year multi-center clinical trial of childhood asthmatics aged 5–12 designed to evaluate the long-term efficacy and safety of inhaled anti-asthma medications. Following completion of the clinical trial, subjects have been reevaluated yearly as a longitudinal follow-up of the natural history of childhood asthma. RNA was obtained from peripheral blood CD4+ lymphocytes collected during a follow-up visit. CD4+ lymphocytes were isolated by positive selection using anti-CD4+ antibody-coated microbeads (Miltenyi Biotec, Auburn, CA). RNA was extracted using the QIAGEN RNeasy Mini Protocol. Expression profiles were generated with Illumina HumanRef8 v2 BeadChip oligonucleotide arrays (Illumina, San Diego CA) according to protocol. Arrays were read using the BeadArray scanner (Illumina) and analyzed using BeadStudio (version 3.1.7) without background correction. Raw expression intensities were processed using the lumi package (Du et al., 2008), with background adjustment with RMA convolution (Irizarry et al., 2003), 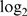 transformation for variance stabilization, and combined-sample quantile normalization. 20,589 transcripts (gene expression traits) were considered for association testing. Adequate DNA for genomewide genotyping was available for all members of 156 parent-child trios of self-reported white ancestry. Genotyping was performed using the Illumina Infinium II HumanHap550 Genotyping BeadChip. Genotyping was performed by Illumina (San Diego, CA) according to protocol. All downstream data analysis was performed locally at the Channing Laboratory. Genotype evaluation and cleaning was performed using PLINK. Marker quality was assessed using a variety of measures including Illumina GC scores, ability to map genomic position of assay sequences to unique sites, parent-offspring genotype incompatibilities, and genotype completion rates.

## Discussion

CCA has the potential to be a powerful tool for identifying relationships between genotype and gene expression. It out-powered pairwise univariate regression in simulations where one SNP affected the expression of one gene when heritability was low. This difference in power was largest under the recessive model with a sample size of 200. Univariate regression with Bonferroni corrected pvalues tends to be overly conservative when heritability is low. Our results suggest that CCA may be useful for locating regions with association under difficult circumstances such as small sample size, small effect size, and recessive genetic models. Furthermore, the simple and intuitive method of picking the SNP with the largest coefficient (disregarding sign) is a fairly well-powered way to recover which SNP was truly correlated among the set of SNPs found significant by CCA.

This study has several limitations. CCA is a method designed to detect linear relationships between normally distributed variables and may not be sensitive to nonlinear relationships between SNPs and gene expression traits. Also, the simulations were based on a single region and only one SNP was associated with a single eQTL. In the data analysis, no gold standard existed for determining which SNPs were truly associated with an eQTL. We focused on applying CCA to SNPs and expression quantitative trait loci (eQTLs) in the same region of the genome; however, this method could straightforwardly be applied to sets of SNPs and eQTLs defined by gene pathways or any other criteria. Furthermore, one could choose one set of eQTLs (or SNPs) and test for canonical correlation with each set of the partitioned regulatory variants (or associated eQTLs).

CCA could also straightforwardly be used as a screening method to find regions of strong regulation and reduce multiple comparisons. For instance, if the data include parental genotypes, then, in a preliminary screening stage, the expected offspring genotypes (given the parental genotypes) could be used in place of the true genotypes in CCA. Promising areas of the genome could then be further tested using FBAT [11]. In this case, the screening stage would not compromise the significance of the second stage since the FBAT conditions on parental genotypes and phenotypes. CCA might also be useful for determining hotspots of genetic regulation and as a way to search for regulatory variants controlling the expression of genes in pathways.

CCA is a method to be considered in genetical genomics studies. It reduces multiple comparisons, is more powerful under some of the scenarios where univariate regression methods are underpowered, and may yield further insight into the true relationships between multiple SNPs and eQTLs by providing canonical variates.

## Supporting Information

Figure S1 Linkage disequilibrium (measured by D′) between 20 tag SNPs. The color red indicates D′ = 1 and a LOD score≥2. Blue indicates D′ = 1 and LOD<2. Pink indicates D′<1 and LOD≥2. White indicates D′<1 and LOD<2.
(2.27 MB TIF)
