# Supplementary figures and images for: Using Canonical Correlation Analysis to Discover Genetic Regulatory Variants
Source: PLoS One. 2010 May 13;5(5):e10395. doi: 10.1371/journal.pone.0010395 (PMC2869348; doi:10.1371/journal.pone.0010395)

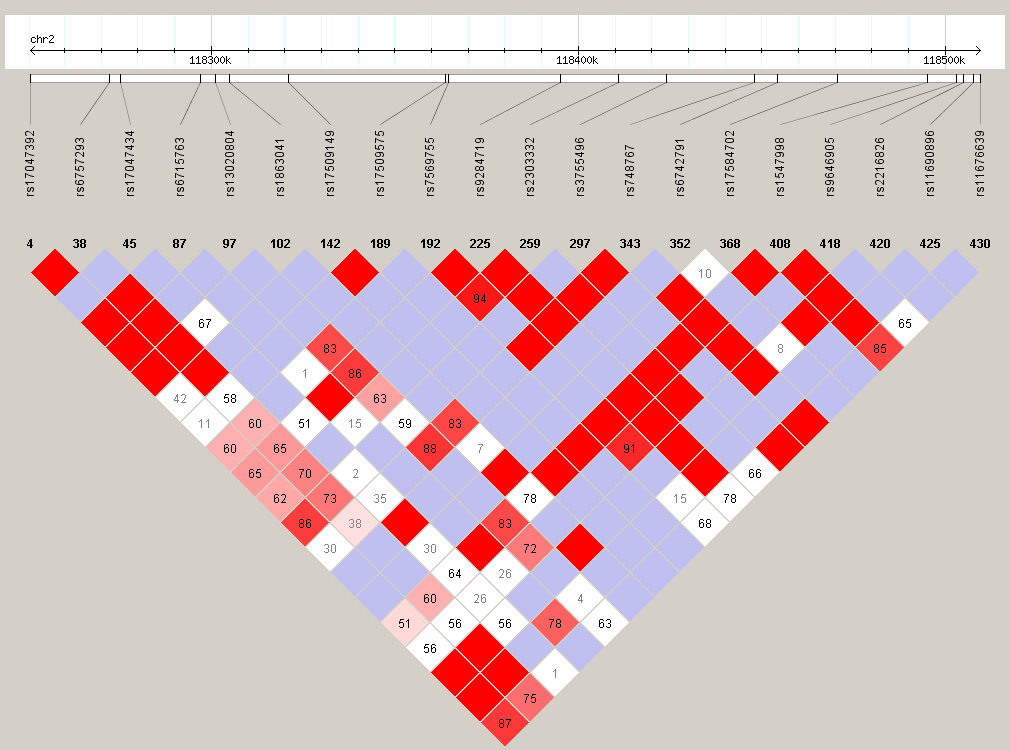

Supplement: Figure S1 — Linkage disequilibrium (measured by D′) between 20 tag SNPs. The color red indicates D′ = 1 and a LOD score≥2. Blue indicates D′ = 1 and LOD<2. Pink indicates D′<1 and LOD≥2. White indicates D′<1 and LOD<2. (2.27 MB TIF) [file pone.0010395.s001.tif]
